# How to build a game for empirical bioethics research: The case of ‘Tracing Tomorrow’

**DOI:** 10.1111/hex.13380

**Published:** 2021-10-29

**Authors:** David M. Lyreskog, Gabriela Pavarini, Jessica Lorimer, Edward Jacobs, Vanessa Bennett, Ilina Singh

**Affiliations:** ^1^ Department of Psychiatry, Warneford Hospital University of Oxford Oxford UK; ^2^ Wellcome Centre for Ethics and Humanities University of Oxford Oxford UK

**Keywords:** big data, coproduction, digital bioethics, mental health, predictive testing, young people

## Abstract

It is becoming increasingly clear that the field of empirical bioethics requires methodological innovations that can keep up with the scale and pace of contemporary research in health and medicine. With that in mind, we have recently argued for Design Bioethics—the use of purpose‐built, engineered research tools that allow researchers to investigate moral decision‐making in ways that are embodied and contextualized. In this paper, we outline the development, testing and implementation of a novel prototype tool in the Design Bioethics Workshop—with each step illustrated with collected data. Titled ‘Tracing Tomorrow’ (www.tracingtomorrow.org), the tool is a narrative game to investigate young people's values and preferences in the context of digital phenotyping for mental health. The process involved (1) Working with young people to discover, validate and define the morally relevant cases or problems, (2) Building and testing the game concept in collaboration with relevant groups and game developers, (3) Developing prototypes that were tested and iterated in partnership with groups of young people and game developers and (4) Disseminating the game to young people to collect data to investigate research questions. We argue that Design Bioethics yields tools that are relevant, representative and meaningful to target populations and provide improved data for bioethics analysis.

**Patient or Public Contribution:**

In planning and conducting this study, we consulted with young people from a diverse range of backgrounds, including the NeurOX Young People's Advisory Group, the What Lies Ahead Junior Researchers Team, Censuswide youth participants and young people from the Livity Youth Network.

## 1 DESIGNING BIOETHICS RESEARCH

Traditionally, the field of bioethics has its background in the construction of theoretical frameworks to be tested and operationalized in practice to a greater or lesser degree. Over the last couple of decades, however, bioethicists have gradually recognized the need for a more multi‐ and interdisciplinary set of methodologies, leading to what is now commonly referred to as ‘the empirical turn’ in bioethics.^1^ This diversification in research approaches has undoubtedly enriched the field, allowing rigorous investigations through theoretical work, as well as empirical work involving quantitative^2^, ^3^ and qualitative^4^, ^5^ methods. However, the methodology of empirical bioethics has so far relied heavily on established methodology of adjacent fields such as psychology and social sciences: surveys, interviews, case studies and so forth. Meanwhile, the same neighbouring fields have enjoyed significant progress through development of new and experimental methodologies. Prominent examples of such tools are videogames and virtual reality scenarios, used to measure constructs ranging from social avoidance^6^ and judgement–behaviour discrepancy,^7^ to problem‐solving skills.^8^

Despite these developments in neighbouring fields, bioethics has yet to embrace technological resources to support robust empirical research. We have^9^ argued that a way forward for bioethics as a field is *Design Bioethics*, which is ‘the design and use of purpose‐built, engineered tools for bioethics research, education and engagement’. Design Bioethics argues for critical and innovative design of empirical tools in bioethics in ways that attend to researchers’ theoretical and epistemological commitments. Technology‐based tools, such as digital games, allow researchers to enact theoretical frameworks within bioethics that point to the importance of context, narrative presence and embodiment in moral decision‐making.^10^, ^11^ The premise of these purpose‐built tools is to improve researchers’ ability to encourage active reflection in participants and to investigate decision‐making in contextualized, emotionally relevant scenarios and narratives, thereby increasing ecological validity in measurement. Such tools could allow researchers to respond to the need for scalability and representativeness in bioethics research, matching the pace and scale of developments in health and medicine.

A key challenge for the bioethics research community is how to design, test and implement novel tools in a systematic and rigorous way. In this paper, we present a method to design a digital tool for bioethics research based on our experience developing Tracing Tomorrow (www.tracingtomorrow.org)—a game created to investigate adolescents′ attitudes and preferences around digital phenotyping in psychiatry.

**Figure 1:**
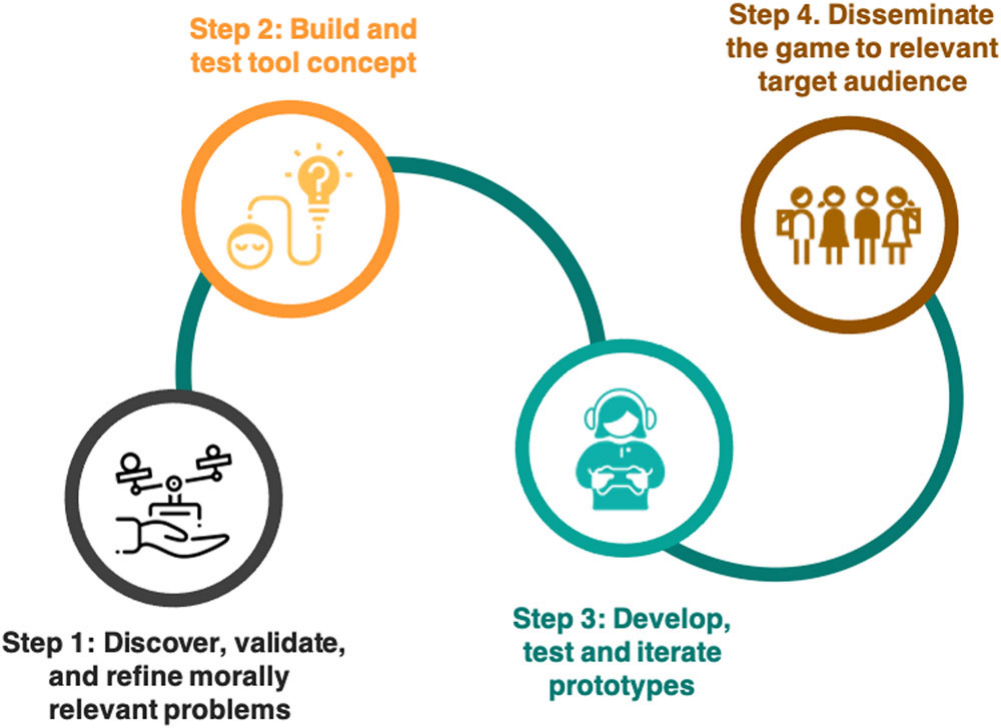
A systematic method of developing a game for bioethics research

In what follows, we provide a structured description of the four stages outlined in Figure 1 using Tracing Tomorrow as a prototype. To provide context, we begin with a brief overview of the theme that motivated Tracing Tomorrow and our rationale for using digital games in this specific case; also, we describe the research team's composition and external collaborators.

## 2 PRELIMINARIES: THE TEAM & THE PROJECT

Tracing Tomorrow is a game developed as part of an interdisciplinary project focused on the ethics of the early intervention (EI) paradigm in psychiatry, which emphasizes the need to identify, prevent and intervene upon risk of poor mental health, ideally before symptoms manifest or become clinically significant. The project analyses ethical concerns that EI may pose for young people with and without mental health diagnoses, particularly in relation to stigma, moral agency and personal identity and normative conceptions of ‘good’ (virtuous) citizenship (www.begoodeie.com).

**Figure 2:**
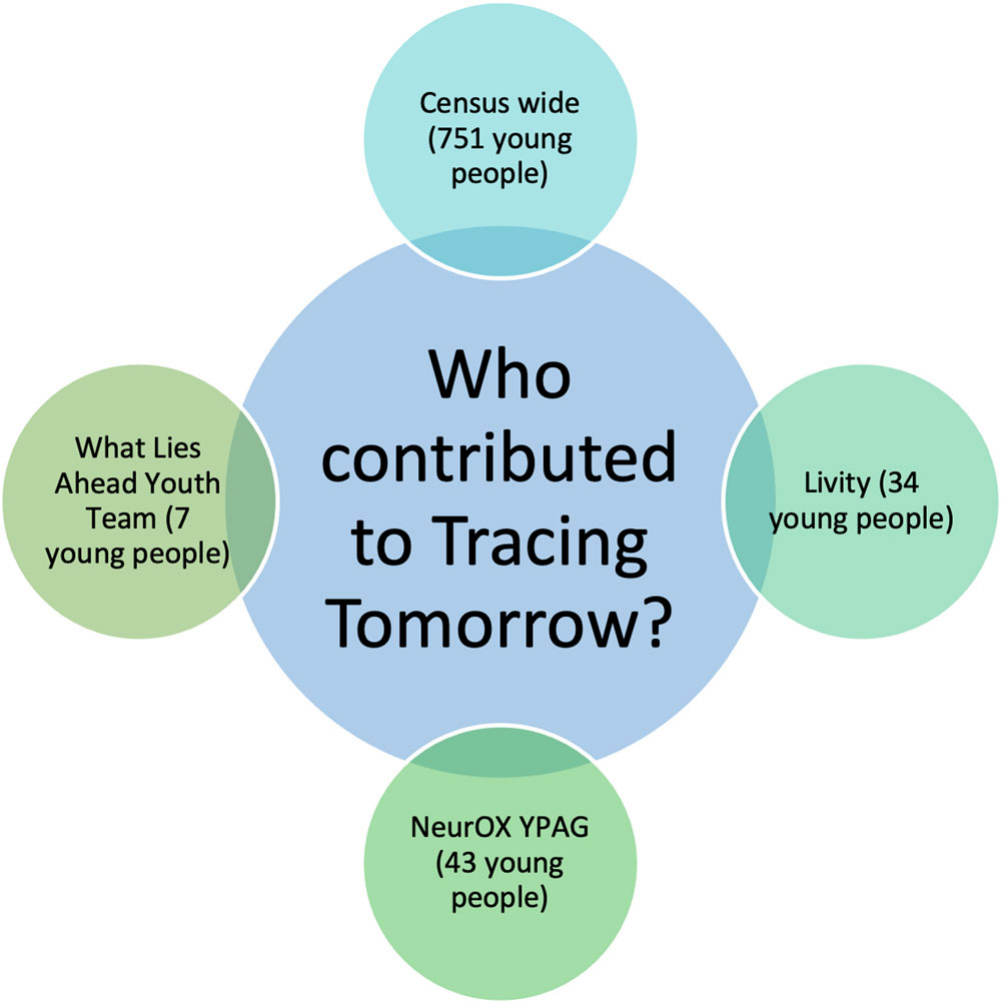
Youth involvement strategy (The NeuroOX YPAG is a group of approximately 45 young people (aged 14–18) that collaborates with researchers on mental health ethics and methods; The What Lies Ahead Youth Team is a team of seven junior researchers from across Europe conducting research in mental health ethics; Censuswide is a research company with an extensive network of young people; and The Livity Youth Network is a group of young people who work as consultants for the London‐based bureau Livity.)

The research team was multidisciplinary, utilizing expertise in bioethics, social psychology, philosophy and public engagement. The aim of the project was twofold: First, to develop a scalable solution to engage and empower young people in informed reflection and decision‐making around innovations in mental health prevention and intervention and second, to conduct research to inform bioethical understanding of young people's attitudes and preferences around digital mental health innovations. The team collaborated with the NeurOX Young People's Advisory Group (NeurOX YPAG); a game design studio (Preloaded, http://preloaded.com); a marketing agency focusing on youth empowerment (Livity, https://livity.co.uk/), and the Department IT team. Our youth engagement strategy entailed consulting with young people from a diverse range of backgrounds (see Figure 2, youth engagement strategy) and followed the principles of coproduction that we have outlined elsewhere.^12^

## 3 STEP 1: DISCOVER, VALIDATE AND REFINE MORALLY RELEVANT PROBLEMS

In this first step of the Design Bioethics process, the aim is to work together with stakeholders to explore and subsequently clearly define research goals, core concepts and a set of ethically charged issues that are both relevant to the stakeholders and significant from a research perspective. As this phase will decide the direction of the entire enterprise, informing every subsequent choice, it is of utmost importance that this foundation is thoroughly researched and established. Ideally, this phase utilizes both qualitative and quantitative methods to ensure capture of core themes and moral problems. It is important to note that this is not a strictly linear process: It is highly unlikely that core themes immediately emerge that are readily defined and of the highest importance to stakeholders and to researchers. Instead, it is a process of iteration and reiteration, exploring ways in which moral problems and ethical issues are understood, experienced and expressed. While it may be tempting to ‘wait and see’, and to leave conceptual issues raised in this phase unresolved, we found that if left aside too long, it became difficult to repair fundamental conceptual and terminological gaps.

In preliminary work with the NeurOX YPAG, we had defined the topic for the game project as digital phenotyping in schools. *Digital phenotyping*—defined here as the collection and analysis of diverse kinds of digital data for mental health risk identification and prevention purposes—is an important area of development in psychiatry, with increasing emphasis on enabling data collection and interventions in school settings for young people.^13^ In collaboration with Livity, we conducted several further consultations with young people to refine our themes and questions. These consultations set the course for the entire project.

### 3.1 Stakeholder consultation: The qualitative component

The consultation process began with four qualitative workshops, reaching thirty‐four 16–18 year olds. The aim of these workshops was to identify what themes within EI ethics were most relevant and appealing to our target audience. We specifically looked into what types of predictive techniques, mental health challenges and ethical considerations young people find most relevant to their lives.

Young people were recruited via youth mailing lists, social media and schools. Each consultation was 3.5 h long and was structured with both creative exercises and group discussions. These consultations were led by two members of Livity, and joined by a member of our research team in the role of an observer, all of whom had experience working with young people. The content of the workshops was centred around our research interests in ethical issues in predictive testing for mental health.

To facilitate young people's engagement with the subject matter, we presented them with a number of different scenarios involving mental health predictions. We asked the young people to comment on how interesting and relatable the scenarios were and on the ethical implications that stood out for them. We also consulted groups on what game format they would find most engaging, and how to ensure that data collected throughout gameplay were valid and meaningful (see Table 1).

**Table 1:** Key findings from the qualitative stakeholder consultation session

Relatability	Participants expressed a preference for playing as a young person—who had themselves received a risk assessment or whose friend had—rather than an adult, e.g., on a school board or in charge of a predictive testing service
	‘I don't like the idea of being an older person in charge of younger people’ (Harriet, 17)
	There was significant support for setting the subject matter in a relatable, realistic context that all users would be familiar with: school, home or the digital environment of their phone
	‘School is just a cliche setting but it's relatable to everyone, everyone gets it’ (Matthew, 18)
	Relatability also drove a preference to explore the ethical consequences of using digital/social media tracking information, rather than results of genetic testing
	‘Everyone can relate to it [social media tracking]’ (Joanna, 16)
	Participants felt it best to focus on one of a smaller number of mental health difficulties that they found particularly relevant—stress problems, anxiety problems and depressed mood—rather than addressing multiple conditions at a more superficial level
	‘I know friends who have told their parents about their anxiety or depression and their parents didn't believe them’ (Tanya, 17)
	‘Anxiety and depression can just be seen as attention seeking, so good to go deeper on these’ (Hasham, 17)
Ethical	Matters of identity—how young people see themselves and are seen by others—and privacy were seen as particularly important when considering digital phenotyping and mental health risk: Digital behaviours online were seen as more deeply personal and private than biomarkers
	‘Privacy is a big thing for young people’ (Isaac, 17)
	Although participants recognized that giving away data is a common occurrence in exchange for access to online content, transparency about data collection and anonymity was seen as crucial
	‘Make it clear it's being used for research that is going to help people… [that] it's anonymous and not being sent to your school or teachers’ (Anna, 17)

### 3.2 Stakeholder consultation: The quantitative component

At the second stage of consultation, a UK‐wide online survey was designed to validate and to expand on the findings from the qualitative section with a larger sample. Data from the qualitative stage were used to create multiple‐choice questions that investigated young people's attitudes and preferences across a number of domains. Key questions the survey investigated included the following: (i) the extent to which young people considered different mental health challenges to be relevant to themselves and/or their friends (on a scale from 1—not relevant to 5—very relevant), ethical issues that they found most appealing to explore in the context of mental health predictions and their preferences for exploring the implications of receiving a risk assessment for themselves versus a close other.

**Figure 3:**
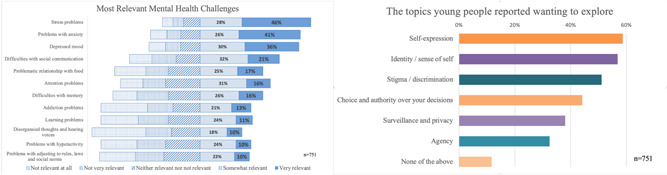
Young people's views on the relevance of different mental health challenges and ethically relevant themes to explore

The survey was completed by a total of 751 young people aged 16–18 years. Depressed mood, anxiety and stress problems were perceived as the most relevant mental health challenges, over memory problems, attention problems and hyperactivity, psychosis symptoms and antisocial personality traits (Figure 3, left). In terms of ethically relevant themes, self‐expression, identity and stigma were the themes that most participants wished to explore (Figure 3, right; participants could tick all options that applied). Young people indicated that their answers would be more truthful if the game explored the implications of mental health risk predictions for themselves, that is, if they were ‘at risk’ (35.8%), rather than a friend, family member or romantic partner (all < 24%).

### 3.3 Wrapping up Step 1

We used the findings from the qualitative and quantitative consultations to specify the main research question for our game: What are young people's values and preferences in the domain of digital phenotyping in schools? Drawing upon the consultations, we worked together with the NeurOX YPAG, Livity and Preloaded to develop a set of research themes to investigate throughout the game. These themes were as follows: (1) *Trust*, (2) *Knowledge & Support*, (3) *Identity* and (4) *Normative disposition*. We developed initial definitions of these themes, which we iterated through communications with the game design team.

*Trust* included expressed preferences for sharing private information and/or data and experienced trustworthiness of diverse sources of support. *Knowledge & Support* designated preferences relating to the extent to which young people value, search for and use information to make informed choices and to seek support. *Identity* sought to capture participants′ self‐understandings and self‐expressions throughout the process of learning more about the risk of mental health challenges. Finally, *Normative dispositions* sought to capture young people's values and preferences for the management of the harms and benefits of digital phenotyping in relation to mental health risk prediction.

## 4 STEP 2: BUILD AND TEST TOOL CONCEPT

Step 2 is to develop an *alpha build* of the tool—an early version of the tool with only fundamental mechanics in place—to ensure that it can collect the data required to pursue the research goals. In this phase, the technical dimensions of the tool; its ability to adequately investigate the research questions; and its societal translation—that is, its uptake potential—should be tested both independently and in combination. For example, one needs to ensure that data can be safely and correctly collected and stored; that scenarios can be crafted to answer specific research questions; and that those scenarios are deemed relevant and interesting to the target population. The team understood this phase to be more focused on tool development itself, rather than on the underpinning research questions and themes. However, we found that in reality, this phase forced us to interrogate our research aims and methods.

The alpha build of the tool cannot proceed without answers to two core questions. First: *What* do you want to find out from participants? Having set the themes of interest and formulated research questions on an abstract level, this is the point where those higher‐level questions need to be translated into questions directed towards the target population. Second: *How* should you ask the questions to best capture authentic and meaningful responses? Of course, these questions also arise in other empirical protocols, such as in projects that use structured interviews or surveys. Purpose‐built engineered research tools present novel challenges because the technology enables the researcher potentially to leverage multiple epistemological positions. With more advanced tools (than the prototype we used for our study), the system can integrate and triangulate data from these positions and use this information to direct further play. One could thereby motivate branching narratives that pursue ever deeper (and possibly more idiosyncratic) dimensions of the research questions. This would be a significant technological advance from the two‐dimensional survey or interview.

For us, however, it was also important at this stage to assess the risk of excessive complexity compromising our aim to capture authentic responses. We chose a game approach to collect data on embedded and contextualized situations in real time. We therefore needed the game to motivate authentic (or nearly so) responses to obtain meaningful data. Ideally, the game would immerse the participant to a point where they were not *hypothesizing* what they might do in a certain situation, but instead, were *acting authentically* in the game. One of the potential tradeoffs, therefore, was the intrigue of the game environment (which would attract players and motivate them to continue play to the end) and the need for players to play authentically as themselves. This tradeoff was exemplified in lengthy discussions about whether or not to allow players to create an avatar to play in the game. Consultations with the NeurOX YPAG indicated that avatar creation was an attractive feature that would help create immersion. However, the risk of allowing the creation of an avatar is that players might make choices according to what they thought that *the avatar* would prefer and value (i.e., roleplaying), rather than what reflected *their own* values and preferences. We investigated the impacts of this tradeoff through user testing and consultations with the NeurOX YPAG by inquiring how young people might experience the game with or without an avatar, respectively. Ultimately, due to concerns about roleplaying risking hindering authentic choice, we made the decision to try to make the game feel engaging to players using different means than an avatar.

As part of the conceptual focus in Step 2, the game studio challenged the researchers to specify what our game would be about. Together, we developed the following game setting:

While preparing for her final exams, a secondary school student receives information that, based on their digital footprints, they are at elevated risk of developing depression; and the student has to deal with the consequences of this information.

**Figure 4:**
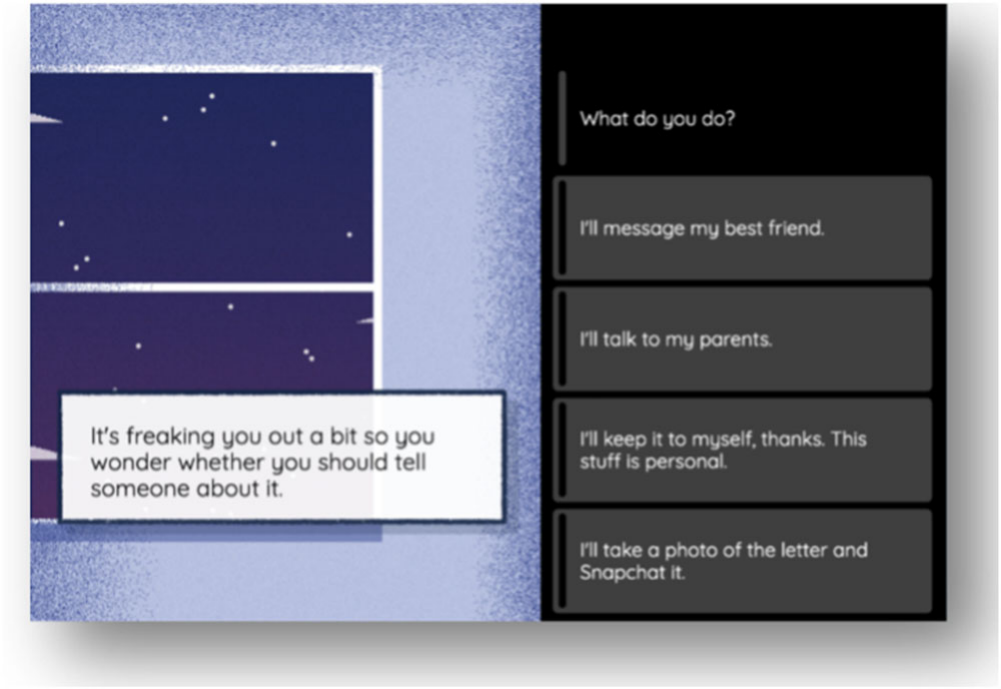
Example choice for the *Trust* theme

**Figure 5:**
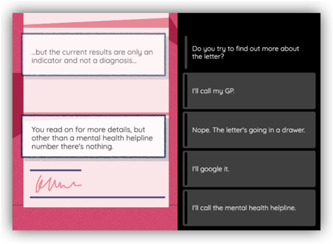
Example choice for the *Knowledge & Support* theme

Faced with information about their risk of depression, the player navigates different choice situations—including practical decisions and moral dilemmas—that emerge throughout a continuous narrative. We tested this general direction and explored a number of potential in‐game choice situations with a group of 30 young people aged 16–18 years during a discussion session hosted on WhatsApp. Using feedback from the session, we next developed a series of choice situations covering the four themes established during Step 1:

(1) *Trust*: The main research question here was as follows: Who—if anyone—do young people trust in handling information about the risk of poor mental health? Our consultations allowed us to generate relevant and convincing options, including sharing information with someone close (e.g., to a parent in private); sharing information publicly in person (e.g., in a group of friends); and sharing publicly online (e.g., posting on social media). In this way, we were able to gather data about how young people view mental health data in relation to networks and relationships (see Figure 4).

(2) *Knowledge & Support*: The central line of inquiry here was as follows: What do young people value and prefer in searching for and using information to make choices and seeking support? For example, do they prefer face‐to‐face or online sources, informal support or help from experts? (Figure 5).

Within this theme, we were also interested in determining the extent to which young people would use information about the themes explored in the game. This line of inquiry inspired the inclusion of optional ‘fact’ icons throughout the game, which young people could click to obtain more information. The inclusion of these icons was motivated by a wish to utilize games’ ability to support informed choice through simple, yet effective mechanisms. An example of such facts includes ‘Internet or mobile tech can be used to collect or monitor signs of mental wellbeing in someone’.

**Figure 6:**
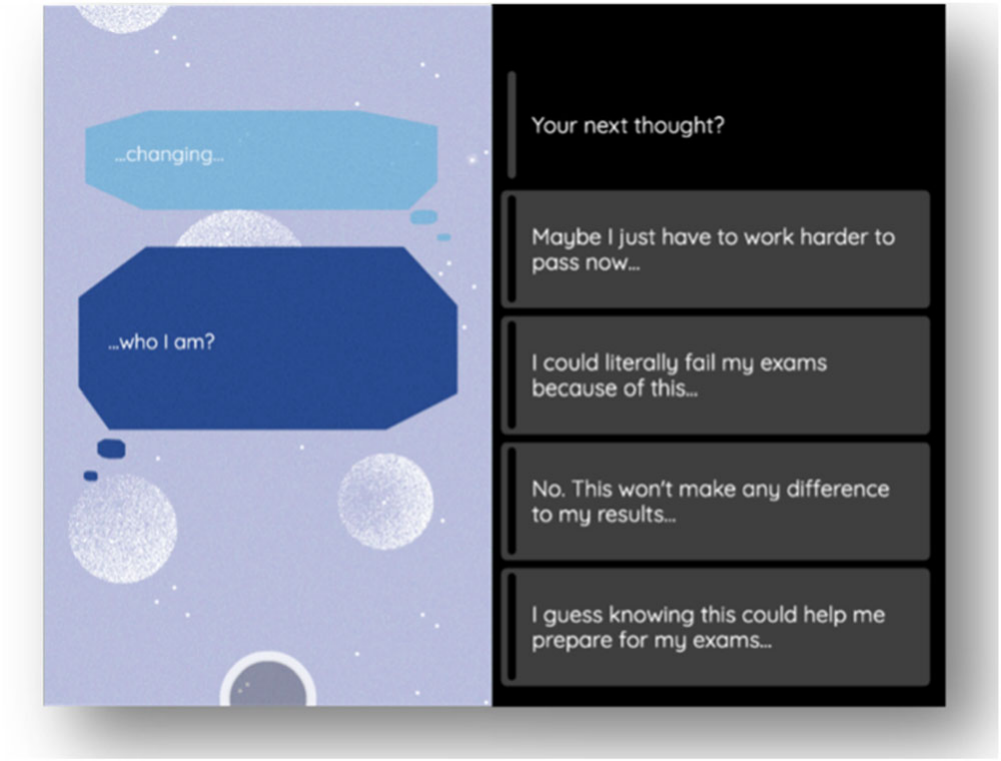
Example choice for Identity theme

**Figure 7:**
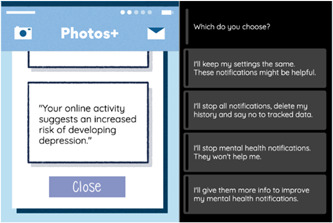
Example scenario for the *Normative disposition* subtheme

(3) *Identity*: Our main line of inquiry under this theme was whether receiving risk information about one's mental health would affect a young person's self‐understanding across a range of domains. We investigated this, for instance, in a scenario where the player‐character had trouble sleeping and started thinking about the prediction and whether this could affect ‘who they are’ and their performance in the upcoming exams (Figure 6).

(4) *Normative disposition*: The main line of inquiry under this theme was what young people's normative disposition towards digital phenotyping for mental health was. We were interested in participants’ attitudes towards schools, health systems and social media platforms performing such assessments as well as their stances on data linkage between schools and health care systems. An example is shown in Figure 7, where, after receiving a letter, the player receives an automated social media notification also stating that they are at risk for depression. The choice alternatives were designed to reflect young people's preferences in relation to the value of privacy and data control in this context.

## 5 STEP 3: DEVELOP, TEST AND ITERATE PROTOTYPES

The third step is to develop prototypes to be tested and iterated in partnership with stakeholders and developers. Dissemination strategies also need to be developed at this stage, so as to align with testing and iterating designs. This process of iterative development is key to the process of Design Bioethics and allows continuous input and involvement from relevant stakeholders to ensure a meaningful and smooth user experience. A challenge here is to build the tool in accordance with the themes and research goals set out in the previous steps, while at the same time remaining responsive to feedback about how exactly to make the tool accessible and attractive to the target populations. It is important that the strategies developed to optimize tool accessibility and engagement align with the dissemination strategies to enhance those very aims. Planning ahead, and thinking about how to bring participants to the tool, will help in designing the tool so that people want to use it once they arrive at it—and vice versa.

In building Tracing Tomorrow, game design took place in parallel with the development of strategies enabling capture of research data from gameplay. Technically, this meant that we needed to transfer participant data from the game interface to the backend for analysis, without interrupting player experience. To achieve this, we needed to understand the different ways in which the game might be played. We conducted testing with pairs of young people, through a process of user observation/observed play. During the course of a 1‐h session, two adolescents at a time would play the game and discuss their experiences. These sessions were led by one facilitator from the marketing agency, and included three observers (one member from the research team and two members from the game developers’ team). In addition to these structured, recorded sessions, the research team also sought feedback on the game prototypes from the NeurOX YPAG and a team of junior researchers who led a related project^9^ via informal group sessions or email.

User testing offered important insights for further game development, touching on the overall ‘feel’ of the game’; design; narrative; relevance; mental health literacy and support; and language. These factors supported the process of designing a game and a user experience that would help us achieve our research aims. We needed gameplay to produce valid and reliable results; we needed players to play through the entire game to obtain complete data from an individual's gameplay; and we needed to attract and retain a diversity of users. Testers suggested modifications to the language to make it sound less like adults trying to mimic teenagers. They thought that the beginning of the game was abrupt, and they were confused about the real aim of the game, noting that their experience was that games tricked the user about the intentions behind the game. Further technical feedback included that the scrolling and tapping features were slow and awkward.

Responding to this feedback, we made a number of amendments, including adding small animations throughout each scene to bring the game to life; improving the game mechanics; and working with the NeurOX YPAG, making the language more age appropriate. To provide context and help players understand the aim of the game, we added a section at the beginning explaining the setting and context of the game, and inviting players to play ‘as themselves’.

## 6 STEP 4. DISSEMINATE THE GAME TO THE RELEVANT TARGET AUDIENCE

The final stage is to disseminate the tool, and to follow up on any issues that may emerge during or after launch. As highlighted earlier, the development of the tool itself and the dissemination strategy should intersect early in the project, with continuous feedback loops that include all project partners.

**Figure 8:**
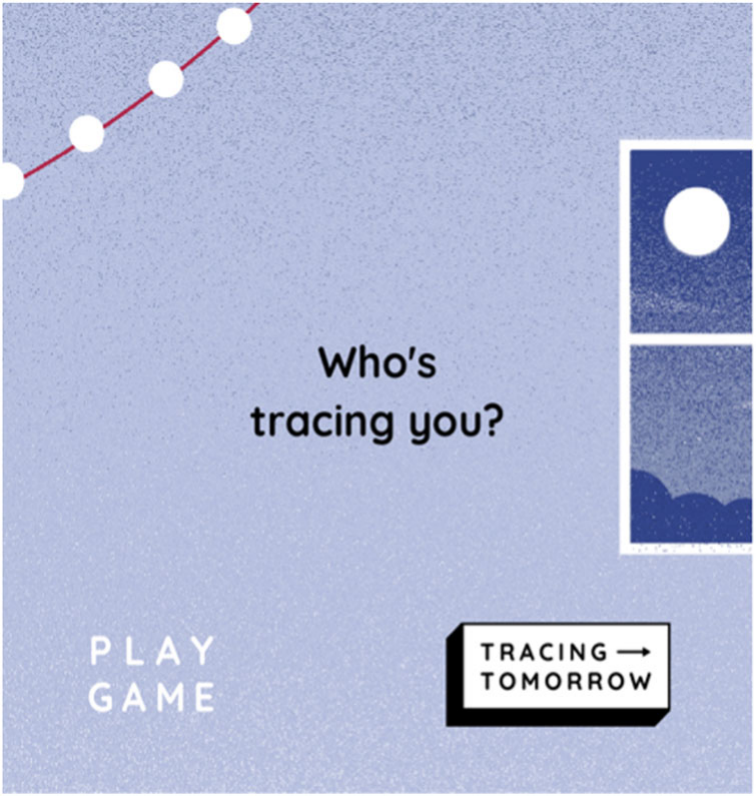
Example of a social media marketing asset

As we developed the research themes and aims for Tracing Tomorrow, we were conducting a parallel process with our marketing agency partner to ensure that the design would also meet our dissemination and engagement objectives. We settled on a 4‐week launch strategy focusing on (1) social media advertisement (Instagram and Twitter); (2) posts by Instagram influencers; and (3) web‐radio appearances. The themes and design of the social media ads were codeveloped with the NeurOX YPAG and designed and produced by the marketing agency (Figure 8).

The influencers whom we chose to work with were selected from a pool of candidates provided by the marketing agency, who researched social media platforms for appropriate channels. Parameters included the size of following (particularly United Kingdom following); audience age range and average (focus on 16–18 year olds); social scope in audience (e.g., ethnic minority representation); experience with, and/or interest in, mental health challenges and related topics. To ensure fit with their specific following, influencers were given the freedom to design their own content, with support from the marketing agency when applicable.

We also utilized web‐radio spots with a radio station aimed at young people in the United Kingdom (Wizard Radio), with sponsored discussions and host–listener interaction sessions about the topic of the game (digital phenotyping in schools) and short plugs for the game itself.

## 7 DISCUSSION

As a field, empirical bioethics has remained largely reliant on ‘traditional’ instruments such as surveys,^2^, ^3^ interviews and the occasional panel^4^, ^5^ to substantiate claims and investigate ethical dilemmas. With a few exceptions,^14^, ^15^, ^16^ empirical bioethics appears to lag behind the methodological curve, as neighbouring fields such as experimental psychology and neuroscience quickly innovate. Methodological innovation should be driven by the desire to improve the evidence base representing specific phenomena, and there are clearly gains to be made in adopting tools to better situate and vividly represent morally charged decisions.^9^, ^10^, ^11^ Moreover, constructing a novel methodology from the bottom up allows the development of purpose‐built tools that can yield richer and more meaningful data sets and enhance engagement with key stakeholders.

The process of developing Tracing Tomorrow was intense, involving close and constant collaboration among the research team, external partners and young people. The final product enabled us to engage young people at scale in research on the ethics of digital phenotyping.

It was important that our academic team was multidisciplinary to enable translation of bioethics concepts into empirical methodology and that industry partners had game design expertise, youth marketing experience and technical resources and support. The involvement of young people in every step of the process, and across the research and design components of the project, was critical to our eventual success. As argued elsewhere,^9^ the continuous inclusion of feedback loops and stakeholder coproduction is not only an integral component of Design Bioethics as an approach to bioethics methodology but also an overall powerful tool to improve recruitment in—and impact of—empirical bioethics research.

Technical support is essential in a project like this, from the concept phase through to dissemination. Having the technical backend of the project supported by technical expertise meant that when we collected data from almost 20,000 players within 4 weeks of launch (as compared to the anticipated 1500 players), our systems were able to support this scale of data collection. A small subset of players clicked through to a website dedicated to the game, where they registered independently for participation in future research studies. This enabled us to capture a research population in accordance with data ethics standards. We decided to locate this click‐through option at the very end of the game so as not to interrupt the game experience.

Tracing Tomorrow was a 2‐year project, costing about £250,000, including game design and marketing; postdoc time; a project manager; and payments for all the young people involved in the project. With a bigger budget, we could have designed a much more interactive experience, and there certainly was a temptation to build in ever‐fancier features. However, budget considerations aside, the young people whom we worked with urged us to stick to a more ‘old‐fashioned’ game experience. Their guidance was that for young people, mental health risk was a serious issue, and it would be jarring and potentially disturbing for our game to go too far down the ‘entertainment’ track. We learned that a bigger budget does not necessarily make for a better game, from a player perspective. This sensitive balance between respect for our topic and our target population and the need and desire to ‘scale up’ our research was perhaps the core challenge that bound us together as a multidisciplinary team.

## 8 CONCLUSION

Tracing Tomorrow was planned and carried out as a prototype project to test Design Bioethics as a valid approach to empirical bioethics. The aim was to showcase digital games as a method to engage and to conduct research with young people. Tracing Tomorrow allowed us to investigate young people's values and preferences on digital phenotyping in the United Kingdom. We built an immersive setting to align with theoretical frameworks in bioethics that point to the importance of narrative, context and embodiment in moral decision‐making.^10^, ^11^ The reach and scale of Tracing Tomorrow point to the value of this approach as part of empirical bioethics research into a rapidly evolving biomedical landscape. Our stepped design and dissemination approach included ongoing attention to ensuring accessibility to and relevance for underrepresented groups. For example, we involved a broad range of young people from diverse sociodemographic and national backgrounds throughout the project (see Figure 1), and our dissemination work with influencers aimed to reach specific groups of young people, including Black and minority ethnic groups, and males. However, because we limited the initial sociodemographic survey that preceded the game start, we do not have rich demographic data on the young people who eventually played the game. In future work, we will capitalize on the lessons learned about access, reach and scale throughout this project, to progress more targeted design and dissemination approaches and to evaluate our success in involving and reaching diverse groups. We consider that reporting on such successes (and failures) is an important dimension of transparent research design in empirical bioethics.

In this paper, we have sought to provide a walkthrough example of how Design Bioethics was used to tailor research methodology and digital innovation for a specific set of purposes. Tracing Tomorrow illustrates a systematic and robust method of developing, testing and implementing digital tools that are relevant, representative and meaningful to the target audience, thereby delivering trustworthy data. Our hope is that our example can support and inspire researchers who wish to contribute to methodological innovation, coproduction and improved evidence in studies on the social and ethical dimensions of novel biomedical interventions.

## CONFLICT OF INTERESTS

The authors declare that there are no conflict of interests.

## AUTHOR CONTRIBUTIONS

David M. Lyreskog and Gabriela Pavarini contributed to the design of the study, data collection and analysis, and writing of the manuscript. Jessica Lorimer and Ilina Singh contributed to the design of the study and to writing of the manuscript. Vanessa Bennett and Edward Jacobs contributed to data collection and analysis, and to writing of the manuscript.

## Data Availability

The data from the quantitative consultation that supported the design of the game are available through the Open Science Framework: https://osf.io/pum69/files.
